# ROCK inhibitors enhance the production of large lipid-enriched 3D organoids of 3T3-L1 cells

**DOI:** 10.1038/s41598-021-84955-7

**Published:** 2021-03-09

**Authors:** Yosuke Ida, Fumihito Hikage, Hiroshi Ohguro

**Affiliations:** Department of Ophthalmology, Sapporo Medical University School of Medicine, Sapporo, Japan

**Keywords:** Cell biology, Molecular biology

## Abstract

Since the recent discovery of prostaglandin-associated peri-orbitopathy, a great deal of interest has developed concerning the side effects of anti-glaucoma medications toward periocular fatty tissue, especially their adipogenesis. Two- or three-dimension (2D or 3D) cultures of the 3T3-L1 cells were employed to elucidate the effects of the Rho-associated coiled-coil containing protein kinase inhibitor (ROCK-i) the anti-glaucoma drug, Ripasudil, and other ROCK-i, such as Y27632 on adipogenesis. Ultrastructure by electron microscopy and physical stiffness measurements by a micro-squeezer demonstrated the 3D organoids had essentially matured during the 7-day culture. The effects of ROCK-i on 3D organoid sizes, lipid staining, the mRNA expression of adipogenesis related genes, *Pparγ*, *Cebpa* and *Leptin,* and extracellular matrix (ECM) including collagen (COL) 1, 4 and 6, and fibronectin, and physical stiffness were then conducted. Upon adipogenesis, the sizes, lipid staining and mRNA expressions of adipogenesis related genes, *Col 4* and *Col 6* were dramatically increased, and were further enhanced by ROCK-i. Micro-squeezer analysis demonstrated that adipogenesis resulted in a marked less stiffed 3D organoid and this was further enhanced by ROCK-i. Our present study indicates that ROCK-i significantly enhanced the production of large lipid-enriched 3T3-L1 3D organoids.

## Introduction

Rho-associated coiled-coil containing protein kinases (ROCKs), members of the serine-threonine protein kinase family, were first recognized as RhoA-binding proteins that regulate the remodeling of the actin cytoskeleton^1,2^. The amino acid composition and the carboxyl termini of ROCK 1 (ROKβ) and ROCK 2 (ROKα) are similar and the catalytic kinase domain and the Rho-binding domain (RBD) are also similar but the coiled-coil region of these proteins have a relatively low homology^3^. Functionally, ROCK isoforms play important roles including the regulation of actin cytoskeleton organization, cytokinesis, differentiation, apoptosis, glucose metabolism, cell adhesion/motility, and inflammation^4–7^. ROCKs are also distributed within the ocular and surrounding tissues, including the trabecular meshwork, ciliary muscles, and the retina^8,9^, and are recognized as being involved in the ocular physiology as well as in the pathogenesis of several ocular diseases including cataracts, retinopathy, and corneal dysfunction^10–15^. These observations, in turn, strongly suggest that ROCKs may be therapeutic targets for these diseases. In fact, ROCK inhibitors (ROCK-i) have recently been shown to reduce intraocular pressure (IOP) in several animal models^16,17^, and in Japan, Ripasudil hydrochloride hydrate (Rip), a non-selective ROCK-i, is currently being used as a new member of the class of anti-glaucoma medications for the treatment of glaucoma and ocular hypertension^18,19^.

Among long-term users of prostaglandin analogues (PGs), several recent reports indicated periocular side effects called “prostaglandin-associated peri-orbitopathy (PAP)”, which include deepening of the upper eyelid sulcus (DUES) and others^20^. As a possible mechanism for causing DUES, it has been suggested that PGs inhibit adipogenesis of the orbital lipid based upon MR imaging findings^21^. Since DUES has become apparent as periocular side effect caused by PGs, the effects of other anti-glaucoma medications toward periocular tissues, especially orbital fat, has attracted considerable interest. In order to elucidate molecular mechanisms for causing DUES by PGs, we recently replicated DUES models using three-dimension (3D) cell cultures of 3T3-L1 cells^22^ or human orbital fibroblasts (HOFs)^23^. The findings indicated that PGs significantly affected their adipogenesis as well as extracellular matrix (ECM) metabolism. Since ROCKs also affect adipogenesis, as evidenced by the in vitro studies as above, further studies of the role of ROCK-i toward adipogenesis in our DUES model is of great interest.

Therefore, in the present study, to report on the effects of the ROCK-is, Rip and Y27632 on the adipogenesis of the 3T3-L1 cells, a most widely used as adipocyte cell line for adipogenesis related research, we studied adipogenesis by lipid staining as well as adipogenesis related gene expressions, their ECM expressions (2D and 3D cell cultures), and sizes and physical stiffness by a micro-squeezer of the 3D organoid. In addition, during the course of the 3D 3T3-L1 organoid culture, their physical properties including the 3D organoid size, appearance by phase contrast and electron microscope images, and stiffness were also investigated.

## Results

The objective of this current study was to evaluate the effects of ROCK-is toward adipogenesis and ECM expression in 3T3-L1 cells. In our previous studies, we found that the effects of PGF2α agonists and EP2 agonists on adipogenesis as well as the expression of ECM in 3T3-L1 cells were significantly different between their 2D and 3D cultures^22,24^, and the findings suggested that 3D cultures may become a reliable ex vivo model for use in this research field. However, our 3D drop-culture method has not yet fully characterized so far, especially in terms of the physical properties, sizes and stiffness of organoids during the period of their culture. Therefore, the effects of ROCK-is, Ripasudil (Rip) and Y27632 on adipogenesis and ECM expression on 2D cultured 3T3-L1 cells were initially examined and these effects were then investigated using 3D 3T3-L1 organoid cultures, in which their physical properties during the course of the culture were also characterized, as described below.

### Effects of the ROCK-i of Ripasudil (Rip) or Y27632 on adipogenesis, and mRNA expressions of major ECMs including collagen (Col) 1, Col 4, Col 6 and fibronectin (Fn) of the 2D cultured 3T3-L1 cells

**Figure 1:**
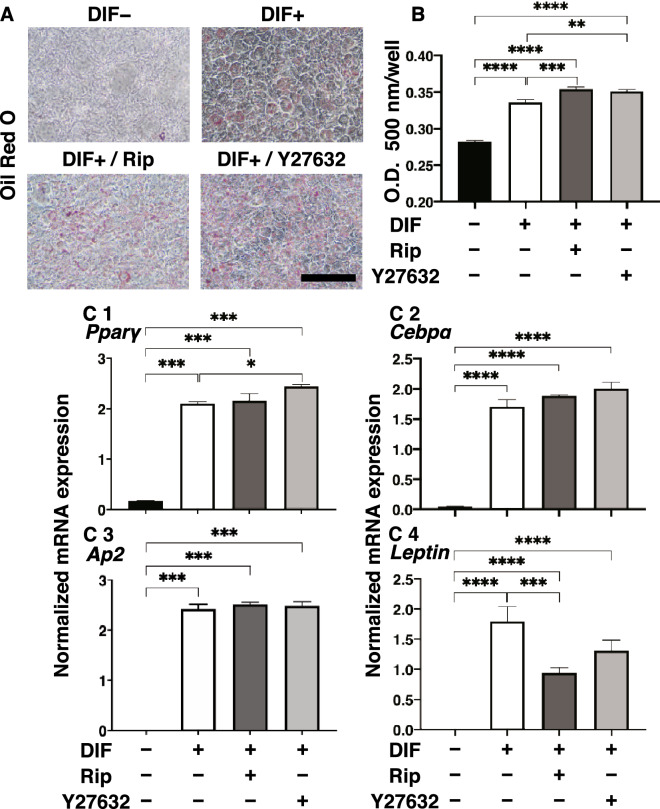
Effects of ROCK-i on adipogenesis of 2D cultured 3T3-L1 cells. 2D cultures of 3T3-L1 cells were performed under several conditions; preadipocytes of 3T3-L1 cells (DIF−) and their adipogenic differentiation (DIF+) with or without 10 µM Ripasudil (Rip) or Y27632. These were stained by Oil Red O (**A**, scale bar: 100 μm) and their staining intensities were plotted (**B**). The mRNA expressions of adipogenesis related genes including *Pparγ*, *Cebpa* and *Leptin* under the above conditions were plotted in (**C**) 1–3. All experiments were performed in triplicate using fresh preparations, each consisted of 5 samples. Data are presented as arithmetic means ± standard error of the mean (SEM). *P < 0.05, **P < 0.01, ***P < 0.005, ****P < 0.001, and other pairs were not statistically significant (ANOVA followed by a Tukey’s multiple comparison test).

**Figure 2:**
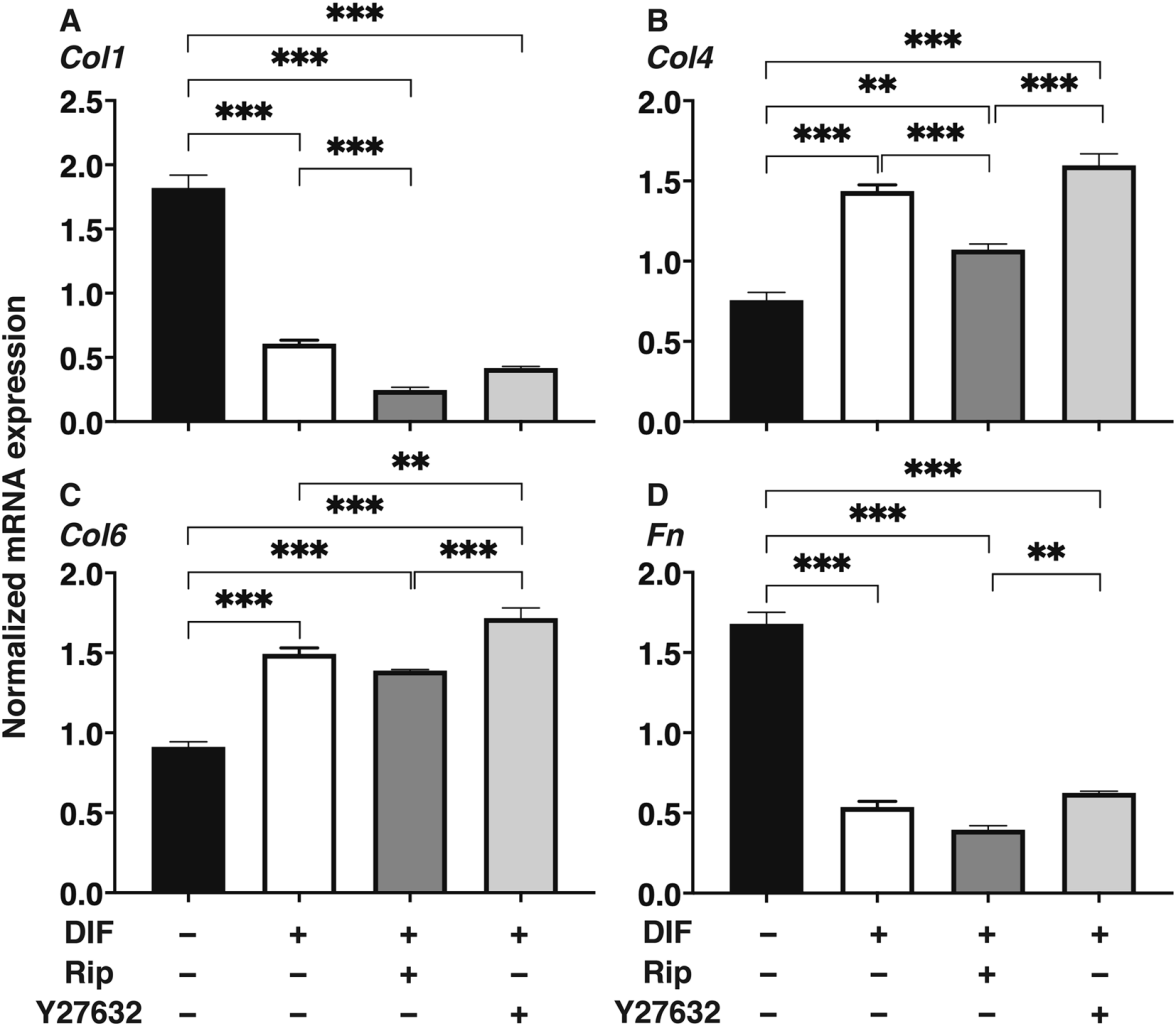
Effects of ROCK-i on ECM expression of 2D cultured 3T3-L1 cells. The 2D cultured 3T3-L1 preadipocytes (DIF−) and their adipogenic differentiation (DIF+) without or with 10 µM Ripasudil (Rip) or Y27632 were subjected to qPCR analysis to estimate mRNA expression of ECMs (*Col1*: collagen 1, *Col4*: collagen 4, *Col6*: collagen 6, *Fn*: fibronectin). All experiments were performed in triplicate using fresh preparations, each consisted of 5 samples. Data are presented as the arithmetic means ± standard error of the mean (SEM). **P < 0.01, ***P < 0.005, and other pairs were not statistically significant (ANOVA followed by a Tukey’s multiple comparison test).

The effects of Rip or Y27632 toward adipogenesis and ECM expression in 2D cultured 3T3-L1 cells were evaluated by lipid staining and quantitative PCR of adipogenesis related genes including *Pparγ*, *Cebpa, Ap2* and *Leptin* and major ECMs components, *Col1*, *Col4*, *Col6* and *Fn*. As shown in Fig. 1, lipid staining by Oil Red O, and the mRNA expression of *Pparγ*, *Cebpa, Ap2* and *Leptin* of the 2D cultured 3T3-L1 cells were significantly increased upon adipogenesis (DIF+). In the presence of 10 µM Rip or Y27632, lipid staining and mRNA expressions of transcriptional regulatory genes, *Pparγ* and *Cebpa*, were further enhanced, while the mRNA expression of the lipid secretory factors, *Leptin*, was significantly decreased, that of the lipid secretory factor, *Ap2* was not altered. In terms of the mRNA expression of major ECMs components of the 2D cultured 3T3-L1 cells, significant down-regulation of *Col1* and *Fn* (panel A and D) and up-regulation of *Col4* and *Col6* (panel B and C) were observed upon DIF+ (Fig. 2). mRNA expressions of the DIF+ induced up-regulated genes (*Col4* and *Col6*) and those of down-regulated genes (*Col1* and *Fn*) were decreased in the presence of 10 µM Rip. While in contrast, in the presence of 10 µM Y27632, both DIF+ induced up-regulated or down-regulated genes expression except *Col1* were increased (Fig. 2). These findings indicated that ROCK-is, Rip and Y27632 have a significant effect on adipogenesis and ECM expression in 2D cultured 3T3-L1 cells, and these effects were similar regarding adipogenesis, but different with respect to ECM expression between the ROCK-is. Since, as stated as above, adipogenesis and ECM expression in 3T3-L1 cells are different depending on their culture status, i.e., for 2D and 3D, and in 3D organoid cultures. We therefore investigated additional features, including the influence of ROCK-was on the physical properties, size and stiffness using a 3D organoid culture system.

### Characterization of physical properties including size and stiffness, and adipogenesis of the 3D cultured 3T3-L1 cells

**Figure 3:**
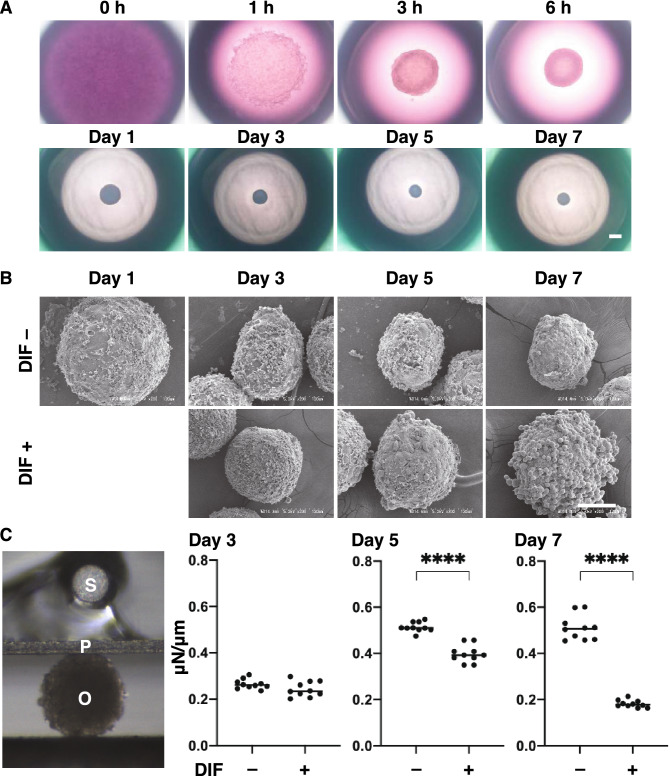
Characterization of the maturation process of the 3T3-L1 3D organoid with (DIF+) or without adipogenesis (DIF−). Representative phase contrast microscopy (PC) images at several time points (0, 1, 3, or 6 h, or Day1, 3, 5 or 7) of 3T3-L1 3D organoids without adipogenesis (DIF−) are shown in (**A**) (Scale bar: 100 µm). Electron microscope (EM) images at several time points (Day1, 3, 5 or 7) of the 3T3-L1 organoids without (DIF−) or with adipogenesis (DIF+) (**B**, Scale bar: 100 µm). Since adipogenic differentiation was induced from Day 1, the EM image of DIF+ at Day 1 is omitted. A single 3D organoid was compressed by a micro-squeezer, and the compression process was continuously monitored by a microscopic camera (**C** left, S: sensor, P: plate, O: organoid). As the physical stiffness at several time points (Day 3, 5 or 7) of 3D organoids without (DIF−) or with adipogenesis (DIF+), the force (μN) required to induce deformation until their half diameter (μm) was reached were measured, and force/displacement (μN/μm) was potted (**C** right). ****P < 0.001, and other pairs were not statistically significant (ANOVA followed by a Tukey’s multiple comparison test).

In advance of the next study using 3D 3T3-L1 organoids, changes in their physical properties, sizes and stiffness were evaluated during the course of the 3D culture. Phase contrast microscopy (PC) demonstrated that the 3T3-L1 preadipocytes (DIF–, approximately 20,000 cells within a 28 μl portion of the 3D organoid culture medium) started to coalesce at 1 h after the seeding, and formed premature organoids by 6 h. Thereafter, the 3D organoid became more apparent and a smaller round shaped mature 3D organoid during Day 1 through Day 7 (Fig. 3A). Electron microscopy (EM) revealed that the 3D organoid adopted a more distinct shape that was clearly surrounded with ECM deposits as their maturation proceeded, and upon DIF+ , the oil droplets became readily apparent (Fig. 3B). These PC and EM images indicated that the hardness of the 3D organoid appeared to increase as to their maturation proceeded, and adipogenesis induced an abundance of oil droplets that may decrease the organoid stiffness. These speculations were entirely confirmed by an analysis of the physical stiffness of the organoids using a micro-squeezer (Fig. 3C). These results indicated that the physical properties, size and stiffness of the 3D 3T3-L1 organoid became evident as above during their 7 day-culture.

### Effects of the ROCK-i of Ripasudil (Rip) or Y27632 on structures and adipogenesis of the 3D cultured 3T3-L1 cells

**Figure 4:**
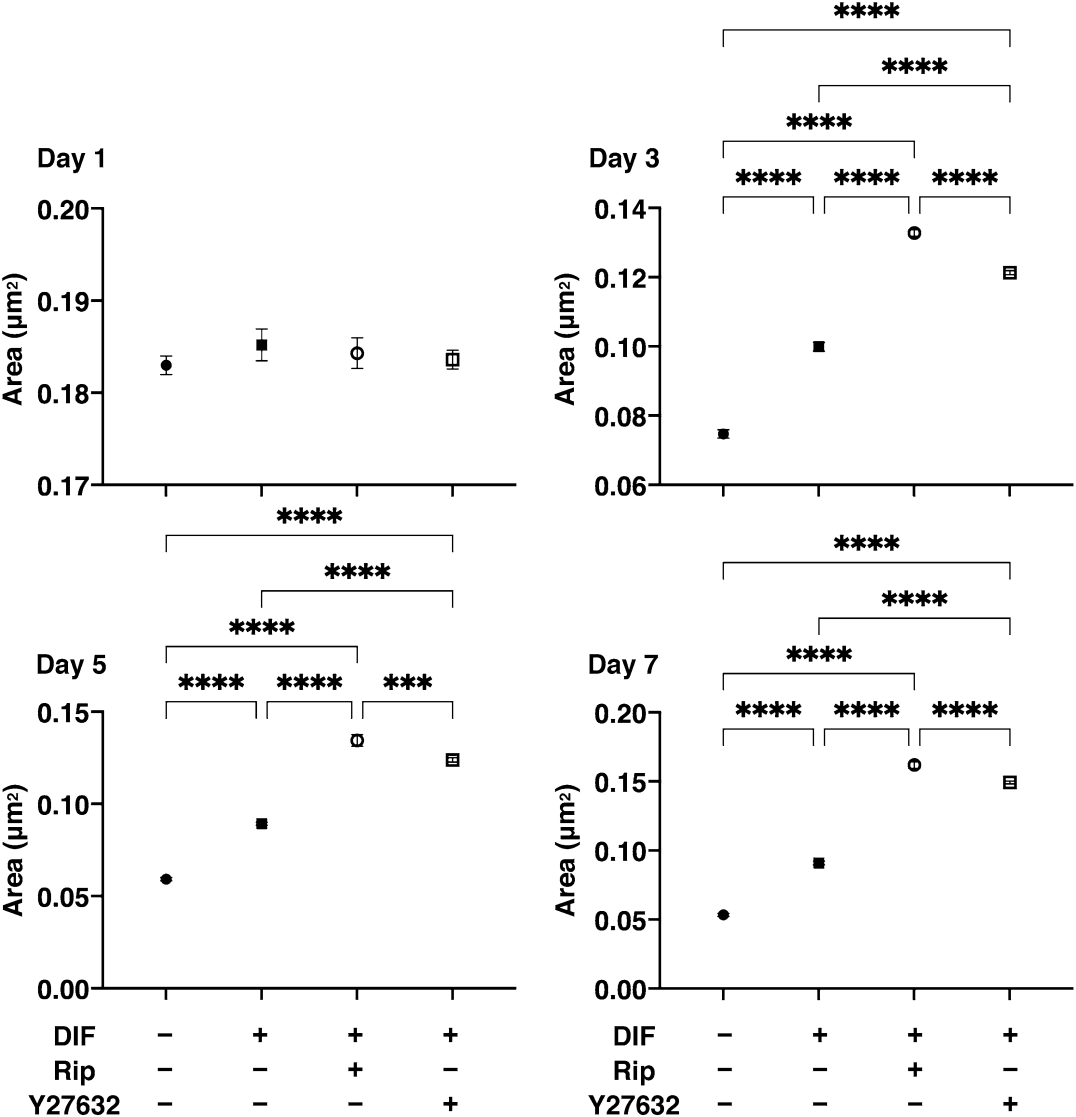
Effects of ROCK-i on area sizes of the 3T3-L1 3D organoids during adipogenesis. The mean area sizes (μm^2^) of the 3D organoids of 3T3-L1 preadipocytes (DIF−, closed circles) and their adipogenic differentiation (DIF+ , closed squares) without or with 10 µM Ripasudil (Rip, open circles) or Y27632 (open squares) were measured. Fluctuations of those during 7-day culture were plotted (left panel) and those at Day 7 were compared among experimental groups (right panel). All experiments were performed in triplicate using fresh preparations, each consisting of 16 organoids. Data are presented as arithmetic means ± standard error of the mean (SEM). ****P < 0.001, and other pairs were not statistically significant (ANOVA followed by a Tukey’s multiple comparison test).

**Figure 5:**
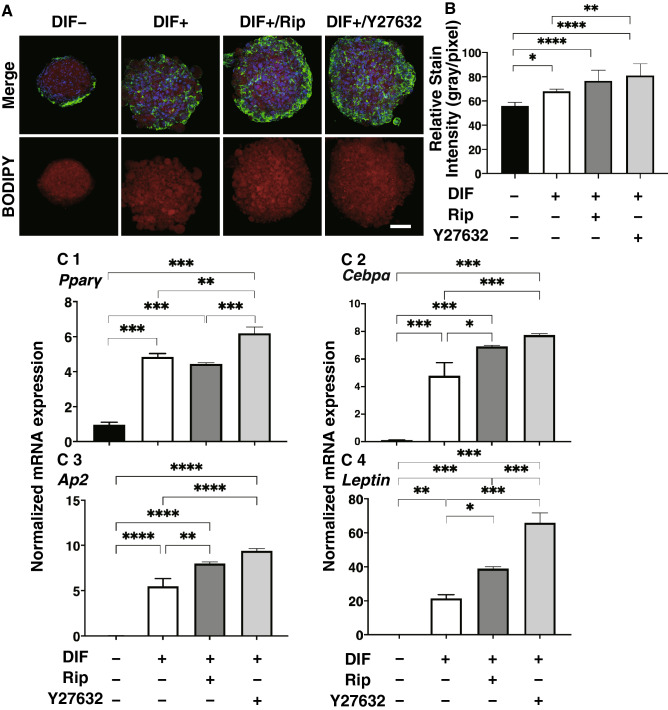
Effects of ROCK-i on adipogenesis of 3T3-L1 3D organoids. 3T3-L1 3D organoids from Day 7 was prepared under several conditions; preadipocytes of the 3T3-L1 cells (DIF−) and their adipogenic differentiation (DIF+) without or with 10 µM Ripasudil (Rip) or Y27632. These samples were immunostained with DAPI (blue), phalloidin (green) and BODIPY (red) (**A**, scale bar: 100 μm) and their staining intensities were plotted (**B**). The mRNA expressions of adipogenesis related genes including *Pparγ*, *Cebpa* and *Leptin* under above conditions were plotted in (**C**) 1–3. All experiments were performed in duplicate using fresh preparations, each consisting of 16 organoids. Data are presented as arithmetic means ± standard error of the mean (SEM). *P < 0.05, **P < 0.01, ***P < 0.005, ****P < 0.001, and other pairs were not statistically significant (ANOVA followed by a Tukey’s multiple comparison test).

Effects of Rip or Y27632 on the structural features of the 3D 3T3-L1 organoids were investigated by measuring their size, and the level of adipogenesis was evaluated by lipid staining and quantitative PCR of adipogenesis related genes including *Pparγ*, *Cebpa*, *Ap2* and *Leptin.* As shown in Fig. 4, consistent with our previous study, uniform round-shape spheroidal organoids of the 3T3-L1 cells were formed and those grew into smaller and matured forms during the 7-day culture. Their mean area sizes were significantly larger upon DIF+ , and such effects by DIF+ were enhanced greatly in the presence of 10 µM Rip or Y27632. In association with the changes in the sizes of areas of the organoids, lipid staining by BODIPY and the mRNA expression of adipogenesis related genes, including *Pparγ*, *Cebpa, Ap2* and *Leptin* of the 3T3-L1 organoids, were also significantly increased upon DIF+ (Fig. 5). In the presence of 10 µM Rip or Y27632, the expression of the transcriptional regulatory gene, *Cebpa* and that for the secretory factors, *Ap2* and *Leptin* were further enhanced (Fig. 5). These findings indicated that ROCK-is induced the formation of larger 3D 3T3-L1 organoids and an enhancement in adipogenesis. To elucidate the underlying mechanisms responsible for causing these RCOK-is’ effects, the mRNA expression of ECM and the stiffness of the 3D 3T3-L1 organoids were evaluated.

### Effects of the ROCK-i of Ripasudil (Rip) or Y27632 on mRNA expressions of major ECMs including collagen (Col) 1, Col 4, Col 6 and fibronectin (Fn) and physical stiffness of the 3D cultured 3T3-L1 cells

**Figure 6:**
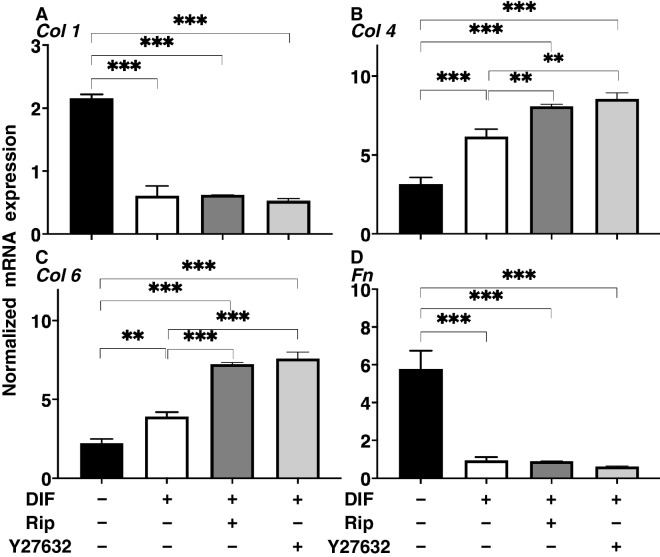
mRNA expression of ECMs in 3T3-L1 3D organoids under several conditions. At Day 7, the 3T3-L1 3D organoids of preadipocytes (DIF−) and their adipogenic differentiation (DIF+) without or with 10 µM Ripasudil (Rip) or Y27632 were subjected to a qPCR analysis to estimate the mRNA expression of ECMs (*Col1*: collagen 1, *Col4*: collagen 4, *Col6*: collagen 6, *Fn*: fibronectin). All experiments were performed in duplicate using fresh preparations, each of which consisted of 16 organoids. Data are presented as arithmetic means ± standard error of the mean (SEM). **P < 0.01, ***P < 0.005, and other pairs were not statistically significant (ANOVA followed by a Tukey’s multiple comparison test).

**Figure 7:**
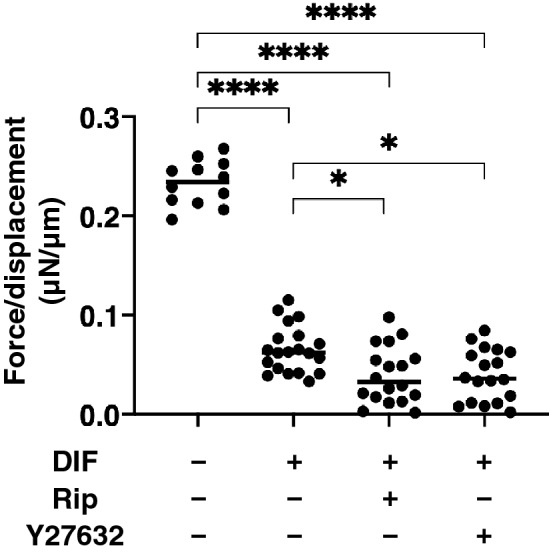
Effects of ROCK-i on physical stiffness of the 3T3-L1 3D organoids. At Day 7, the 3T3-L1 3D organoids of preadipocytes (DIF−) and their adipogenic differentiation (DIF+) without or with 10 µM Ripasudil (Rip) or Y27632 were subjected to a physical solidity analysis by a micro-squeezer. The force required to induce deformation until half diameter was reached (μN/μm force/displacement) were measured and potted. All experiments were performed using fresh prepared 12–20 organoids. *P < 0.05, ****P < 0.001, and other pairs were not statistically significant (ANOVA followed by a Tukey’s multiple comparison test).

The effects of Rip or Y27632 on ECM expression, and the physical stiffness of the 3D 3T3-L1 organoids were evaluated by quantitative PCR of the major ECM components, *Col1*, *Col4*, *Col6* and *Fn*, and a micro-squeezer analysis, respectively. As shown in Fig. 6, a significant down-regulation of *Col1* and *Fn* (panel A and D), and up-regulation of *Col4* and *Col6* (Panel B and C) were observed upon DIF+ as similar to the results from the 2D culture experiments for the mRNA expressions of ECMs (Fig. 2). While in contrast, the ROCK-i induced effects in the 3D 3T3-L1 organoid were different from those for the 2D cultured 3T3-L1 cells, as shown above. That is, mRNA expressions of the DIF+ induced up-regulated genes (*Col4* and *Col6*) were increased and those of down-regulated genes (*Col1* and *Fn*) were not affected in the presence of 10 µM Rip or 10 µM Y27632. Such discrepancy of the effects of the ROCK-is on the ECM expressions between 2D (Fig. 2) and 3D (Fig. 6) cell cultures, which was also recognized in our precedent study may presumably be ascribed to the spatial changes of their ECM expressions in the 3D but not 2D culture^22^. In fact, the ROCK-is induced up-regulated genes, *Col4* and *Col6*, are basement ECM protein, and thus those may be related to the 3D organoid structure. Taken together, these findings indicate that ROCK-is induced a significant enlargement of the 3D 3T3-L1 organoids with a more abundant lipid content and network derived from collagens (*Col4* and *Col6*), in which the former and the latter appear to be related to physical stiffness and stability, respectively. This rationale was confirmed by a physical stiffness analysis using a micro-squeezer. As shown in Fig. 7, the 3D 3T3-L1 organoid became less stiffness upon DIF+ as above, and these effects were further enhanced in the presence of ROCK-is.

## Discussion

A 3D organoid culture was recently been used to stimulate a variety of human pathological conditions^25^. For this purpose, a conventional 2D organoid culture technique conjugated with synthetic scaffolds such as collagen hydrogels^26^, or a liquid-based droplet spheroid culture were employed^27^. Based upon the latter methodology, our group recently developed 3D cultures using a 318-well drop culture plate and successfully obtained 3D organoids from several cells^22,23,28^. In the present study, we characterized the formation and maturation of 3D organoids of the 3T3-L1 cells during the 7-day culture (Fig. 3). Phase contrast microscope (PC) images demonstrated that the 3T3-L1 cells that were dispersed within a 28 μl 3D organoid culture medium formed immature 3D organoids within 6 h after the seeding, and then developed into matured organoids. As examination of the ultrastructure by electron microscopy (EM) revealed that the ECM surrounding the 3D organoid formed a network during Day 5 and oil droplets then became apparent at Day 7. Consistent with this ECM network formation, a micro-squeezer analysis indicated that the 3D organoid stiffness increased and reached a plateau by Day 5. These observations that both an ECM network and adipogenesis maturation reached completion by Day 7 are the rationale for this study using the 3D organoid culture method. These findings also confirm the validity of our recent studies using this 3D organoid from human orbital fibroblast (HOF) cells^23,28^ and 3T3-L1 cells^22^.

Several lines of evidences also indicate that ROCKs are negative regulators of adipocyte differentiation^29^. In terms of 3T3-L1 cells, it was reported that ROCK 2, but not ROCK 1, is responsible for the suppression of the adipogenesis, and in turn, their inhibitors, Y-27632 and fasudil have been identified to promote adipocyte differentiation^29^. In the current study, another pan ROCK inhibitor, Rip also enhanced adipogenesis of the 2D and 3D organoids of the 3T3-L1 cells. Again, such a ROCK-i induced enhancement of lipid staining intensities was clearly evident in the 3D organoid cultures (approximately 10–15% increase) compared to the 2D culture (approximately a 4–5% increase) as shown in Figs. 1 and 5. As a possible reason for this difference between the 2D and 3D culture, we speculate that 3D cultures have considerably more space available for adipogenesis of the 3T3-L1 cells, as compared to the 2D culture. In terms of the mRNA expression of the adipogenesis related genes, *Pparγ*, *Cebpa*, and *Leptin*, were increased upon adipogenesis in both 2D and 3D cultures. However, some differences were observed in terms of the effects of ROCK-i on the expression of these genes between 2D (enhancement of *Pparγ*, *Cebpa*, and suppression of *Leptin*) and 3D culture (enhancement of *Cebpa* and *Leptin*, and suppression of *Pparγ*) (Figs. 1 and 5). There were also some differences between Rip and Y27632 in terms of their efficacies toward the of these genes (Figs. 1 and 5). In addition, those different effects were also varied between adipogenesis regulatory genes, *Pparγ* and *Cebpa*, and secretory hormone, *Leptin*. This may be attributed to differences in their preference for ROCK 1 or ROCK 2 inhibitory activities (Rip; ROCK 1 < ROCK 2, Y-27632; ROCK 1 > ROCK 2)^30,31^, in addition, direct effects toward adipogenesis as well as indirect effects by modulating 3D organoid architectures.

There are many reports concerning the expression of COL1, 4, and 6, and FN in adipocytes or adipose tissues and it has been reported that they are modified during adipogenesis^32–34^. It has been reported that a main type of adipose ECM is the main fibril-forming COL1 and microfibrillar COL6. Collagens (COLs) are triple helical proteins that are present in the ECM and at the interface between cell and ECM^35^. There are various types of COLs and COL-related proteins^36^. Among the COLs, the fibril-forming COL subfamily includes COLs1, 2, 3, 10 and 11, and its structure includes an uninterrupted triple helical domain of approximately 300 nm^35^. COL1 is the most abundant protein and comprises the bulk of COL fibrils, which serve to impart stiffness to tissues and organs^37^. COL4 and 6 belong to network-forming COLs which are involved in the regulation of fibril assemblies and organization as well as integrating cells and matrix structures and/or the integration of different matrix structures such as basement membranes^38^. COL4 networks form the backbone of the basement membrane, and also interacts with basement membrane proteoglycans and glycoproteins that determine its function^38,39^. COL6 binds a large number of extracellular molecules including: COLs1, 2, 9 and 16, microfibril-associated glycoprotein (MAGP-1), FN, integrins and others^40,41^. Based upon these findings, COL6 has the capacity to organize the 3D tissue architecture, which may influence cell migration, differentiation and apoptosis/proliferation to maintain of tissue homeostasis^42^. FN has a weak molecular conformation that can be changed by the binding of allosteric partners or strain resulting from cell contractile forces^43^. The expression of ECM changes characteristically during in vivo and in in vitro adipogenesis^44^. Previous studies using a 2D or 3D cultures of 3T3-L1 preadipocytes revealed the in vitro remodeling from COL1- and FN-rich ECM in preadipocyte cells into further basal membrane type-rich ECMs, such as COL4 and 6, in adipocyte cells^22,32–34^. In the current study, we again confirmed the down regulation of COL1 and FN, and the upregulation of COL4 and COL6 expression upon adipogenesis in both 2D and 3D cultures of the 3T3-L1 cells. In terms of the effects of ROCK-i toward these ECMs expressions, ROCK-i enhanced the adipogenesis-induced upregulation of COL4 and COL6, which was more evident in the 3D organoids than in 2D cultured 3T3-L1 cells, but had no effect on the expression of COL1 and FN. Since COL4 and COL6 are known to belong to network-forming COLs as above, such ROCK-i induced effects toward COL4 and COL6 may facilitate the enlargement of the 3D organoids of the 3T3-L1 cells. Similarly, it has been recognized that the inhibition of ROCK reduces the extent of mechanical tension and stiffness in cells, and decreases ECM synthesis and rigidity in various cell types including the trabecular meshwork^45^.

The molecular mechanisms causing the ROCK-i induced enlarged and less stiff 3D 3T3-L1 organoid remained to be elucidated. As shown in Fig. 5, in the presence of ROCK-i, no cell death and cell proliferation occurred since significant increase or decrease were not detected in the series of sections of the 3D organoid stained by DAPI cell nuclear staining.

Our analysis of the physical stiffness of the single 3D organoid most likely reflects amounts of lipid contents as well as structure foaming proteins including ECMs. Since our result indicated ROCK-i significantly enhanced adipogenesis and basement membrane related ECMs; Col4 and Col6, but not other major ECMs; Col 1 and Fn. Therefore, we speculated that ROCK-i mainly induced increase of lipid contents and simultaneously, basement membrane related ECMs also increase to foam an enlarged 3D organoid to contain such enriched lipid. However, in order to prove this speculation, additional study using inhibitions at different signaling by several specific inhibitors or Si-RNA will be required as our new project.

Recently, a great attention has been paid concerning the periocular side effects of anti-glaucoma medications on orbital adipocytes^46,47^. Since Rip is used as an anti-glaucoma medication, our present data using 3T3-L1 cells suggested that Rip may also affect toward the orbital adipocyte. However, it was revealed that the physiological properties of human orbital fatty tissues are different from those of 3T3-L1 cells. Therefore, effects of ROCK-is to human orbital fibroblasts (HOFs) rather than 3T3-L1 cells will be investigated as our next project.

## Materials and methods

### Adipocyte culture and differentiation of 3T3-L1 cells

To obtain 3T3-L1 organoids, 3T3-L1 preadipocytes (#EC86052701-G0, KAK) grown in two-dimension (2D) cultures until reaching confluence at 37 °C in a 2D growth medium (HG-DMEM containing 8 mg/L d-biotin, 4 mg/L calcium pantothenate, 100 U/mL penicillin, 100 μg/mL streptomycin (b.p. HG-DMEM), and 10% CS) were then subjected to our recently described hanging droplet spheroid three-dimension (3D) culture system^29^. Briefly, approximately 90% confluence of the cells cultured in 100 mm or 150 mm dishes were washed with a phosphate buffered saline (PBS), detached using 0.25% Trypsin/EDTA and resuspended in an organoid medium (2D growth medium supplemented with 0.25% w/v Methocel A4M). Approximately 20,000 cells in the 28 μL organoid medium were placed into each well of the drop culture plate (defined as 3D/Day 0). On every following day, 14 μL of the organoid medium was substituted with a fresh 14 μL organoid medium in each well.

### Adipogenic differentiation of 2D or 3D cultured 3T3-L1 cells with or without ROCK inhibitors (ROCK-i)

The induction of adipogenic differentiation was processed in the 2D growth medium (2D culture) of the organoid medium (3D culture) supplemented with 250 nM dexamethasone, 10 nM T3, 10 μM troglitazone, and 1 μg/ml insulin for two days, and thereafter in the 2D or 3D culture medium supplemented with 10 μM troglitazone and 1 μg/ml insulin. To study the efficacy of ROCK-i, 10 µM Ripasudil (Rip, a generous gift from the Kowa Company Ltd., Nagoya, Japan) or Y27632 (Sigma-Aldrich, St Louis, MO) were added during the adipogenic differentiation.

### Histocytology

Oil Red O lipid staining of the 2D cultured 3T3-L1 cells and BODIPY lipid staining of the 3D 3T3-L1 organoids were performed as described previously^24^. To quantify their staining levels, measurements of optical density of the extracted Oil Red O dye, and the fluorescence intensity of the BODIPY-stained lipid droplets were used, respectively^24^.

### Real-time PCR

The RNA extracted from 16 organoids of each groups were processed to reverse transcription, and each respective gene expression was quantified by real-time PCR as described previously^24^. In terms of assay specificity for qPCR, to guarantee a high quality of the assay, predesigned primers shown in the supplemental Table 1 from Integrated DNA Technologies, Inc. Iowa USA, were used.

### Micro-indentation force measurement

The micro-indentation force of the organoids was measured using micro-squeezer (CellScale, Waterloo, ON, Canada) as described previously^29^. Briefly, a single organoid placed on a 3-mm × 3-mm plate was compressed to achieve a 50% deformation during 20 s under monitoring by an equipped micro-camera. The required strain (μN) was measured, and force/displacement (μN/μm) was calculated.

### Statistical analysis

All statistical analyses were performed using Graph Pad Prism 8 (GraphPad Software, San Diego, CA). To analyze the difference between groups, a grouped analysis with two-way analysis of variance (ANOVA) followed by a Tukey’s multiple comparison test was performed. Data are presented as arithmetic means ± standard error of the mean (SEM).

## Supplementary information

Supplementary information.
