# Supplementary material for: ROCK inhibitors enhance the production of large lipid-enriched 3D organoids of 3T3-L1 cells
Source: Sci Rep. 2021 Mar 9;11:5479. doi: 10.1038/s41598-021-84955-7 (PMC7943807; doi:10.1038/s41598-021-84955-7)
Supplement: Supplementary file 1 — Supplementary information. [file 41598_2021_84955_MOESM1_ESM.pdf]

ROCK inhibitors enhance the production of large lipid-enriched 3D organoids of 3T3-L1 cells

Key words: deepening of the upper eyelid sulcus (DUES), 3T3-L1cell, ROCK, Rho-kinase, ROCK inhibitor, 3-dimension (3D) tissue culture

Yosuke Ida, Fumihito Hikage, Hiroshi Ohguro.

Departments of Ophthalmology, Sapporo Medical University School of Medicine

All correspondence should be addressed to Fumihito Hikage

Tel# 81-11-611-2111, Fax# 81-11-613-6575, e-mail: fuhika@gmail.com

Both authors (Y. I and F. H.) contributed equally to this manuscript.

Gene	Forward primer (5' to 3')	Reverse primer (5' to 3')	TaqMan probe (5' to 3')
**Mouse**			
Pparγ	CTGCTCCACACTATGAAGACAT	TGCAGGTTCTACTTTGATCGC	/FAM/AGCTGACCC/ZEN/AATGGTTGCTGATTACA/IABkFQ/
Cebpα	ACAAGAACAGCAACGAGTACC	TCATTGTCACTGGTCAACTCC	/FAM/CGCAAGAGC/ZEN/CGAGATAAAGCCAAAC/IABkFQ/
Ap2	AAATCACCGCAGACGACAG	CCTTTCATAACACATTCCACCAC	/FAM/ TGAAGAGCA/ZEN/TCATAACCCTAGATGGCG/IABkFQ/
Leptin	GTGCCTATCCAGAAAGTCCAG	AATGAAGTCCAAGCCAGTGA	/FAM/ACCGACTGC/ZEN/GTGTGTGAAATGTCA/IABkFQ/
Col1a1	CGCAAAGAGTCTACATGTCTAGG	CATTGTGTATGCAGCTGACTTC	/FAM/CCGGAGGTC/ZEN/CACAAAGCTGAACA/IABkFQ/
Col4a1	TCTGGCTGTGGAAAATGTGA	AATCCAATGACACCTTGCAAC	/FAM/TCTTTCTCC/ZEN/CTTTTGTCCCTTCACGC/IABkFQ/
Col6a1	CCAGATGAGTGTGAGATCCTG	AAGTTCTGTAGGCCAATGCTC	/FAM/ACCCATTGA/ZEN/CATCCTCTTCGTGCTG/IABkFQ/
Fn1	GAGCTATCCATTTCACCTTCAGA	TTGTTCGTAGACACTGGAGA	/FAM/CAGGAGATT/ZEN/TGTTAGGACCACGGCA/IABkFQ/
36b4	TTATAACCCTGAAGTGCTCGAC	CGCTTGTACCCATTGATGATG	/FAM/AGGCCCTGC/ZEN/ACTCTCGCTT/IABkFQ/

Supplementary Table 1

Sequences of primers and Taqman probes are shown.
